# Unsupervised KPIs-Based Clustering of Jobs in HPC Data Centers

**DOI:** 10.3390/s20154111

**Published:** 2020-07-23

**Authors:** Mohamed S. Halawa, Rebeca P. Díaz Redondo, Ana Fernández Vilas

**Affiliations:** 1 Business Information Systems Department, Arab Academy for Science Technology and Maritime Transport, Cairo 11799, Egypt; 2 Information & Computing Lab, AtlanTTIC Research Center, Universidade de Vigo, 36310 Vigo, Spain; rebeca@det.uvigo.es (R.P.D.R.); avilas@det.uvigo.es; (A.F.V.)

**Keywords:** high-performance computing, time series analysis, unsupervised learning, clustering

## Abstract

Performance analysis is an essential task in high-performance computing (HPC) systems, and it is applied for different purposes, such as anomaly detection, optimal resource allocation, and budget planning. HPC monitoring tasks generate a huge number of key performance indicators (KPIs) to supervise the status of the jobs running in these systems. KPIs give data about CPU usage, memory usage, network (interface) traffic, or other sensors that monitor the hardware. Analyzing this data, it is possible to obtain insightful information about running jobs, such as their characteristics, performance, and failures. The main contribution in this paper was to identify which metric/s (KPIs) is/are the most appropriate to identify/classify different types of jobs according to their behavior in the HPC system. With this aim, we had applied different clustering techniques (partition and hierarchical clustering algorithms) using a real dataset from the Galician computation center (CESGA). We concluded that (i) those metrics (KPIs) related to the network (interface) traffic monitoring provided the best cohesion and separation to cluster HPC jobs, and (ii) hierarchical clustering algorithms were the most suitable for this task. Our approach was validated using a different real dataset from the same HPC center.

## 1. Introduction

High-performance computing (HPC) systems are known for their costly operation and expensive complex infrastructure [1]. Companies and research centers are increasingly demanding this technology to solve different complex computational problems. This has led to a growing need for constant monitoring of HPC systems to ensure stable performance. These monitoring systems are periodically checking the computational nodes of the HPC system to gather the values of different performance counters known as key performance indicators (KPIs) [2]. This information illustrates the operational status of the system. KPIs are usually organized in different categories, regarding the parameters that are being monitored: CPU usage, memory usage, network traffic, or other hardware sensors. Each KPI is often recorded as a time series: different values of the same parameter (KPI) that are periodically gathered, with a specific frequency. Thus, KPIs are usually recorded as a time series matrix that can be processed for different purposes: anomaly detection, optimal resource allocation, visualization, segmentation, identifying patterns, trend analysis, forecasting, indexing, clustering, etc. For instance, abnormal behavior in KPIs may explain or predict the existence of some problems like application issues, work overload, or system faults in the HPC systems. 

Therefore, time series analysis techniques are relevant for the analysis of KPIs. In fact, there are different approaches in the literature [3,4] based on the analysis of a large number of time-varying performance metrics. These proposals apply different techniques, such as statistical analysis [5], machine learning [6,7], and time series [8]. Among all these approaches, machine learning (ML) stands out in analyzing time series data. The availability of the current advanced ML techniques can quickly process a massive matrix with diverse data types, like text, numerical data, or categorical data. These approaches face some common challenges to analyze the gathered data:

- Large data volume. Each HPC node generates a large number of KPIs (usually more than a thousand). Thus, selecting the most appropriate set of KPIs for job analysis is a key aspect [9].
- Large data dimensionality. The KPI matrix that corresponds to one job may contain a huge number of vectors depending on the number of parallel nodes required during its execution.
- Lack of annotated data. This entails problems in validating the models and methodologies. This problem has been highlighted in previous proposals [10], where only a reduced number of annotated KPIs were used. Consequently, the obtained results cannot be considered complete or representative [10,11].

Our research work focused on identifying groups of similar jobs. Since similar jobs tend to have similar performance, we opted to analyze the KPI data obtained from the monitoring system: each job ran in some parallel nodes, and the monitoring system was gathering the KPI data per node. We decided to apply clustering techniques to the information given by the KPIs. Besides, the lack of annotated data drove our research to the application of unsupervised techniques, such as partition and hierarchical clustering algorithms. 

As previously mentioned, the large data volume is an important challenge when analyzing the KPIs. So, one of our objectives was identifying which metrics (KPIs) are the most appropriate for clustering. For this to be possible, we did a two-step analysis. First, we performed clustering by combining KPIs information. Second, we performed clustering using each KPI information individually. The evaluation was done using a real dataset obtained from the Centro de Supercomputación de Galicia (CESGA) (Galicia, Spain, https://www.cesga.es/en/home-2/).

Consequently, our contributions were: (i) a clustering-based methodology that was able to identify groups of jobs that were executed in HPC systems; (ii) simplifying the computational problem by analyzing the different KPIs in order to determine which ones were the most suitable for this type of clustering; (iii) providing the best clustering algorithm in order to identify different types of HPC jobs according to their performance. This methodology could be applied in any HPC to obtain clusters that identify the different types of running jobs. Finally, the resulting clusters constituted the base for a further analysis that would enable the identification of anomalies in jobs. To the best of our knowledge, this approach entailed a novelty approach because of the following aspects: the variety of the KPIs used for our analysis (CPU usage, memory usage, network traffic, and other hardware sensors) and the approach of applying principal component analysis (PCA) reduction in order to face an overwhelming and challenging clustering of KPIs.

This paper is organized as follows. Section 2 presents some background about the techniques used in this research. Section 3 describes the latest work related to time series clustering and anomalies detection in HPC. Section 4 describes the methodology used in this study. Section 5 defines the experiments and their evaluation. Section 6 provides results discussion, and Section 7 covers the conclusions and future work proposals.

## 2. Background

There are three types of learning in ML: supervised, semi-supervised, and unsupervised learning. In supervised learning, the data used for analysis is labeled (annotated) before applying any supervised techniques. One example would be a data table with a sequence of behaviors that have labels. This data table is fed to the supervised algorithm to build a model from the labeled data. This model will be used afterward for future predictions. In semi-supervised learning, part of the data is labeled, and the other is not. Finally, in unsupervised learning, the data is not labeled. For example, an unlabeled data table with a sequence of behaviors is fed to an unsupervised algorithm to group the data with similar behaviors with the aim of labeling these groups later [9].

Since we were dealing with a huge number of KPIs that are not labeled, we decided to consider unsupervised learning techniques and discard other approaches, like classification. In fact, we used clustering techniques that were considered appropriate to discover hidden patterns or similar groups in our dataset without the need for labeled data. In the following subsections, we have introduced the algorithms and the distances we selected (Section 2.1), as well as the different options for clustering validation that helped us to find out the optimal number of clusters (Section 2.2). Finally, we also explained how to deal with a large amount of data by using dimensionality reduction techniques (Section 2.3).

### 2.1. Clustering Algorithms

Clustering algorithms can be classified into five types: partitioning, hierarchical, density-based, grid-based, and model-based methods. Since we were interested in applying clustering to a lower-dimensional time-series (described in Section 4.3), we decided to select partitioning (k-means) and hierarchical (agglomerative clustering) techniques for clustering as they were the most appropriate for this type of data and widely used for our purpose: 

K-means is the most widely used clustering technique, thanks to its simplicity. It partitions the data into K-clusters by enhancing the centroids of the clusters and assigning each object in the data to only one cluster. K-means use the Euclidean distance to measure the distance between all the objects and the corresponding centroids to form the cluster [12]. The main advantages of K-means are that it is simple to implement, it is relatively fast in execution, it can be applied in numerous applications that involve a large amount of data, and it obtains very reliable results with large-scale datasets [13,14].

Strategies of hierarchical clustering are divided into two types: divisive and agglomerative. Divisive clustering is a “top-down” approach where all objects are initially grouped into one cluster. Then, the objects are split gradually into different clusters until the number of clusters equal to the number of objects. Conversely, the agglomerative clustering is a “bottom-up” approach where each object is assigned to an individual cluster at the initial step of the observation. Then, the clusters are progressively merged until they become one cluster. Agglomerative clustering uses a combination of (i) a linkage method [15,16] and (ii) a distance metric to merge the clusters. In our analysis, we used the metrics Euclidean [17], Manhattan [18], and Cosine [19], as well as the following linkage methods:

- Ward’s method. It links clusters based on the same function as the K-means (Euclidean distance).
- Single-linkage method. It links clusters based on the minimum distance between two objects of different clusters.
- Complete-linkage method. It links clusters based on the maximum distance between two objects of different clusters.
- Average-linkage method. It links clusters based on the average distance between all the objects of two different clusters.

Hierarchical clustering has important advantages, such as having a logical structure, setting the number of clusters is not required in advance, it provides good result visualization, and it provides dendrogram-based graphical representation [14,20].

### 2.2. Cluster Validation

Many clustering algorithms require the number of desired clusters as an input parameter. Therefore, the experience of the data analyst and/or the specific requirements of the application of the algorithm are keys in determining that number. However, the cluster validation methods are useful to measure the quality of the clustering results and, consequently, to identify the optimal number of clusters. Clustering validation techniques can be classified into two categories: (i) external clustering validation and (ii) internal clustering validation. The former requires predefined data labels to evaluate the goodness of the cluster, while the latter does not require predefined data labels to evaluate the goodness of the cluster [21]. The KPIs of the HPC jobs are usually unlabeled. Consequently, the internal clustering validation methods are the best option to evaluate the clusters under these circumstances. In fact, our analysis uses three popular internal clustering validation methods to evaluate our clusters: The Silhouette coefficient [22], the Calinski–Harabasz index [21], and the Davies–Bouldin index [23]. These three methods consider their decision the compactness of the clusters and the separation between them. 

The Silhouette index measures the difference between the distance from an object of a cluster to other objects of the same cluster and the distance from the same object to all the objects of the closest cluster. The silhouette score stretches between two values: -1 and 1. The closer the value is to one, the better the shape of the cluster [22]. In fact, a Silhouette score above 0.5 is considered a good result, and a result greater than 0.7 is evidence of a very good clustering [24]. Thus, this technique focuses on assessing the shape or silhouettes of the different identified clusters. Besides, the score obtained with this index only depends on the partition, not on the clustering algorithm [22].

(1) $$s\left(i\right)=\frac{b\left(i\right)-a\left(i\right)}{\max\{b\left(i\right);a\left(i\right)\}}$$

The Calinski–Harabasz index is also identified as a variance ratio criterion, where a cluster validation function is based on the average of the sum of the squared distances among clusters and among objects within the cluster [21]. It focuses on assessing the dispersion of objects within their cluster and the distance from other clusters.

(2) $$CH=\frac{SS_{B}}{SS_{W}}\times \frac{\left(N-K\right)}{\left(K-1\right)}$$

where 𝑁 is the total number of samples, 𝑆𝑆_B_ and 𝑆𝑆_B_ are the between and within-cluster variances, respectively, 𝑘 is the number of clusters.

Finally, the Davies–Bouldin index is used to calculate the separation between the clusters. It focuses on comparing the centroid diameters of the clusters. The closer the Davies–Bouldin value is to zero, the greater the separation is between clusters since zero is the lowest value [23].

(3) DB=1K∑k=1K Suk+ulduk,ulk≠lmax

where S(u_k_)+S(u_l_) is the distance within the cluster, and d(u_k_,u_l_) is the distance between the cluster.

### 2.3. Dimensionality Reduction

HPC KPIs data is usually organized into high-dimensional matrices, which affects the accuracy of any machine learning algorithms and slows down the model learning process. Hence, it is essential to implement a feature dimension reduction technique that combines the most relevant variables in order to obtain a more manageable dataset [25]. There are several techniques used for dimensionality reduction, such as principal component analysis (PCA) [26], t-distributed stochastic neighbor embedding (t-SNE) [27], and uniform manifold approximation and projection (UMAP) [28].

The principal component analysis (PCA) [26] is one of the most widely used methods to reduce data dimensionality. Its goal is to reduce data with a large dimension into a small number of the so-called principal components. These principal components highlight the essential features of real data and are expected to maintain the maximum information (variance) of the original data. There are two approaches to apply PCA: (i) fixed PCA and (ii) variable PCA. In the former, the number of principal components is fixed beforehand, whereas, in the latter, the number of principal components is calculated during the process by analyzing the percentage of variance that is maintained.

PCA has been successfully applied in different research areas [29,30,31,32,33]. However, some of them have revealed two downsides [25,27]. On the one hand, in large dimension covariance matrix, the estimation and evaluation tasks are challenging. On the other hand, PCA mainly focuses on the large invariance instead of the small invariance except for the information that is explicitly given in the training data. However, our analysis did not face any of these problems. The maximum dimensionality of the analyzed jobs in our dataset (described in Section 4.2) was 43 parameters. This made the calculation of the principal components feasible with a percentage of retained information greater than 85% for 80% of the jobs (see Section 4.3).

## 3. Related Work

The increasing demand for HPC technology entails that maintaining the quality of the service is key in data centers. Clustering is one of the techniques that is becoming more relevant for this purpose. Analyzing and comparing the differences and similarities of jobs that are run in HPC systems open the door to further and deeper studies, such as anomalies detection. In fact, security and performance go hand by hand. In fact, Zanoon [34] confirmed this direct relationship between security and performance by analyzing the quality of service of cloud computing services (jobs running in HPC systems). The author concluded that better security meant better and better performance.

In the specialized literature, there are different approaches that focus on clustering the KPIs in order to support the comparison between jobs [6,12,35]. Yahyaoui et al. [12] obtained a good clustering result with a novel approach to cluster performance behaviors. They used different clustering algorithms: K-means, hierarchical clustering, PAM, FANNY, CLARA, and SOM after reducing the dimensionality of time-oriented aggregation of data with the Haar transform.

Li et al. [36] achieved a higher accuracy score for clustering by proposing a robust time series clustering algorithm for KPIs called ROCKA. This algorithm extracted the baseline of the time series and used it to overcome the high dimensionality problem. Besides, Tuncer et al. [35] proposed a new framework for detecting anomalies in HPC systems by clustering statistical features that retained application characteristics from the time series. On another hand, Mariani et al. [37] proposed a new approach named LOUD that associated machine learning with graph centrality algorithms. LOUD analyzed KPIs metrics collected from the running systems using machine learning lightweight positive training. The objective was two-fold: to detect anomalies in KPIs and to reveal causal relationships among them. However, this approach did not work properly with high precision.

## 4. Methodology

**Figure 1:**
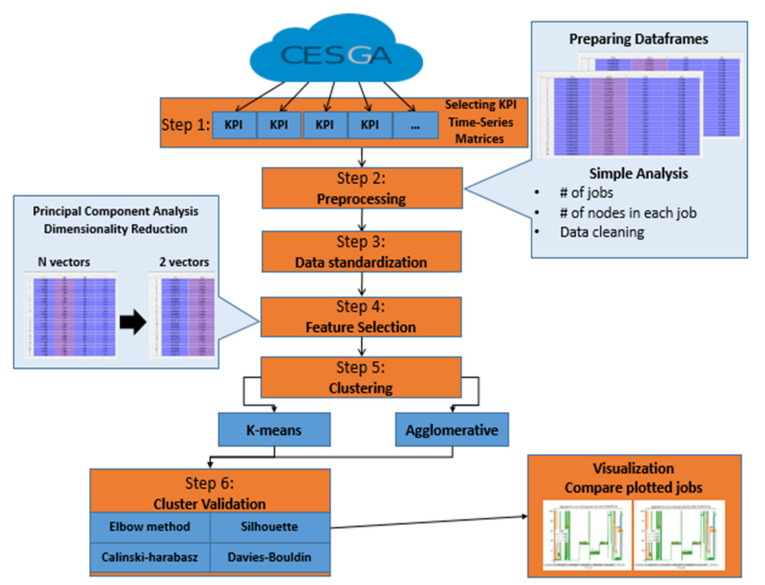
Framework for clustering high-performance computing (HPC) jobs key performance indicators (KPIs) using feature selection.

HPC systems execute a huge number of jobs every day, which is usually done on hundreds of parallel nodes. These nodes are monitored by more than a thousand KPIs. The goal of this study was to identify clusters of HPC job performances based on the information given by their KPIs. We assumed that this task was going to give relevant information about the usual behavior of the jobs, which would be used in the short-term to identify anomalies in jobs. However, this goal brought challenges like data scaling and dimensionality that we faced, defining a six-step methodology, which is summarized in Figure 1. 

The first step was the selection and definition of the KPIs used in clustering (Section 4.1). The second step was data preprocessing (Section 4.2), where we managed to read the data and identify the jobs that were used in operational jobs, which are those that have a systematic nature like scheduled system update, sensors checks, and backups. On the other hand, non-operational jobs are those that have a non-systematic nature. In addition, a basic analysis of non-operational jobs gave us a better view of the data to prepare them for the pre-clustering phase.

Some of the dimensionality reduction methods applied like PCA were affected by the scale, which is a requirement for the optimal performance of many machine learning algorithms. For this reason, a third step to standardize data was needed (Section 4.2). The fourth step was to overcome the dimensionality problem (Section 4.3), always present when analyzing large time-series data, like in our case. The PCA dimensionality reduction method helped to reduce our KPIs matrix and speed up the clustering process. The fifth step was clustering (Section 4.4). Two clustering experiments were performed using K-means and agglomerative hierarchical algorithms with different linkage methods and distance metrics (Section 5). The first experiment clustered the PCAs of the non-operational jobs for all the metrics (KPIs) combined. The second experiment clustered the PCAs of the non-operational jobs for each KPI individually. The study did not have a predetermined number of clusters (K). Therefore, in the sixth step, both algorithms clustered the data considering different values of K (from 2 to 200). Then, the clustered results of all K values were evaluated using three previously mentioned internal cluster validation methods (Silhouette analysis, the Calinski–Harabasz index, and the Davies–Bouldin index) to determine the goodness of the clusters and to identify the optimal number of clusters. The clustering results from both experiments were compared to identify which KPIs showed the best clustering results and, consequently, were the most representative to clustering the jobs. Lastly, a validation experiment was conducted with a new dataset to validate the obtained results.

### 4.1. Performance Data Selection

The execution of HPC jobs is deployed over a high number of nodes, thousands of parallel nodes that are closely monitored by specific systems. As previously mentioned, these monitoring systems are periodically gathering the values of specific metrics or KPIs. Depending on the monitoring system, the information may be overwhelming with thousands of metrics or KPIs. The collected data is stored as a time series matrix per node. These KPIs are usually classified into five different categories:

- Metrics about CPU usage, such as the time spent by a job in the system, owner of the job, nice (priority) or idle time.
- Metrics of the network (interface) traffic, such as the number of octets sent and received, packets and errors for each interface.
- IPMI (intelligent platform management interface) metrics that collect the readings of hardware sensors from the servers in the data center.
- Metrics about the system load, such as the system load average over the last 1, 5, and 15 minutes.
- Metrics of memory usage, such as memory occupied by the running processes, page cache, buffer cache, and idle memory.

For our analysis, we acquired a dataset from the CESGA Supercomputing Center (Centro de Supercomputación de Galicia). Foundation CESGA is a non-profit organization that has the mission to contribute to the advancement of Science and Technical Knowledge, by means of research and application of high-performance computing and communications, as well as other information technologies resources. The dataset stores information about a total amount of 1783 jobs (operational jobs and non-operational jobs), which were running in the 74 available parallel nodes from 1 June 2018 to 31 July 2018.

The collected data gave information about 44,280 different KPIs. In order to filter this overwhelming amount of data, we did a previous filter according to the needs of the CESGA experts. Therefore, we focused our attention on the 11 KPIs summarized in Table 1. The selected KPIs belonged to the five previously mentioned categories (CPU usage, memory usage, system load, IPMI, and network interface) and were selected by the CESGA experts based on their relevance and clear representation of the performance of jobs from each category.

Each KPI gave a matrix with the following information: (i) the value of the KPI, (ii) the time of the machine when the value was acquired, (iii) the job, and (iv) the node to which this value belongs.

### 4.2. Data Preprocessing and Standardization

The objective of this preprocessing phase was to read and organize the KPI matrices into data frames before applying any machine learning steps. For this task, we used the functionality of the Python Pandas library [38]. Additionally, we also did analysis and data visualization that helped understand the nature of our dataset before applying any further analysis, whose results are summarized in Table 2. 

From a total of 1783 jobs, 200 were excluded from our clustering analysis because of one of the following reasons:

- The jobs were not included in all the 11 KPIs matrices, i.e., we did not have complete information about the metrics of the job.
- The jobs were executed in only one node, which entailed they were not parallelized jobs, which was mandatory for our proposed method dimensionality reduction phase.
- These one-node jobs (12% of the dataset) were mostly operational jobs, which were not the focus of our study.
- The analysis of one-node jobs (operational) deserved a specific study that was out of the scope of this paper.

Before proceeding to job clustering, we split the 1583 jobs into two types: operational totaling 1281 jobs and non-operational totaling 302 jobs. As it was previously mentioned, our analysis focused only on non-operational jobs. Consequently, we ran two clustering experiments considering the 302 non-operational jobs. In the first experiment, clustering the 11 KPI matrices combined and, in the second experiment, clustering each KPI matrix individually.

Table 3 shows the number of nodes per non-operational job in our dataset. The executable nodes count per job revealed the following: zero jobs were executed on only one node, 195 jobs were executed on less than 5 nodes, and 49 jobs were executed on nodes in between 6 and 10. Finally, the calculation showed that 80.7% of the jobs were executed on less than 10 nodes.

The standardization process is usually a required step before applying any machine learning algorithm in order to achieve reliable results [39]. In our case, we proceeded to do this standardization stage because PCA was affected by scale, and the values gathered in the 11 KPI matrices ranged from very low to very high values. Thus, the data was standardized into a unit scale: the mean was equal to zero, and the variance was equal to one. 

### 4.3. Jobs KPIs: Dimensionality Reduction

One of the major challenges in KPIs analysis is the large volume of available data. After pre-processing our dataset, each column of the matrix represented the KPIs of the nodes that were being used to run the jobs in parallel. The number of nodes was proportional to the parallelization and computational needs of each job as (Time × Nodes) matrix. Analyzing our data, we could see that 19.3% of the jobs were executed on more than 10 nodes. We also had the time series storing the KPIs for each node, so the analysis of such volume of data was overwhelming. Consequently, we decided to apply a dimensionality reduction method to overcome this challenge. As previously mentioned, we decided to use PCA to reduce the dimensionality of the matrix that represented the KPI gathered data of each job. The objective was reducing this dimensionality without losing information (variance) and, therefore, reducing the computation load and execution time of the clustering algorithms. 

We decided to apply a fixed PCA technique with two principal components. This decision was based on two aspects. On the one hand, our initial analysis (Section 4.2) showed that 195 jobs of the total had from two to five nodes. Moreover, 80.7% of the jobs were executed on less than 10 nodes. Thus, applying more than two principal components did not seem to be appropriate in this context. On the other hand, we checked that applying two principal components was enough to retain information (variance) of the original data (job KPIs performance): the percentage of retained information was greater than 85% in 81% of the jobs, as Table 4 shows.

The PCA was applied to each KPI matrix individually, resulting in a matrix of (time × 2 principal components) for each job. On the one hand, for experiment one (Section 5.1), we used jointly the information of the 11 KPIs. For this, we took advantage of the Python Pandas library [38] to combine and flatten the PCA results of each job for the all-11 KPIs into one row in a data frame labeled with the job number, resulting in a matrix of (jobs × (times × 2 principal components × KPIs)). Each row in this data frame represented the PCAs for all 11 metrics combined with each job indexed by job number. On the other hand, for experiment two (Section 5.2), we analyzed each KPI individually. Thus, the PCA results of each job for each KPI were combined and flattened into one row in a separate data frame labeled with the job number, resulting in a matrix of (jobs × (times × 2 principal components).

### 4.4. Clustering

**Figure 2:**
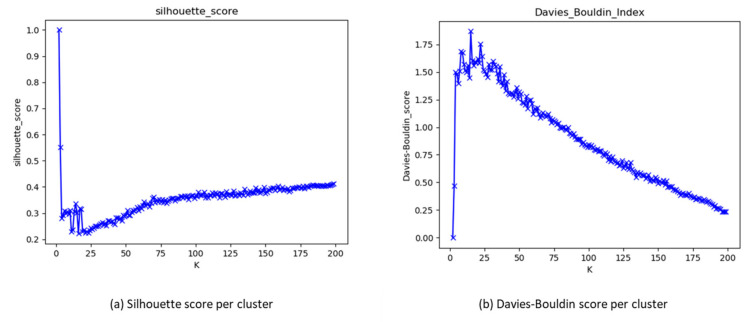
Clustering quality scores: (**a**) using the Silhouette score and (**b**) using the Davies-Boulding score.

The study applied the K-mean algorithm and the agglomerative hierarchical algorithm to cluster the jobs for both experiments. On the one hand, the K-means used only Euclidean distance for clustering. On the other hand, the agglomerative hierarchical algorithms used three distance metrics—Euclidean, Manhattan, and Cosine—with different linkage methods for clustering. Both algorithms were applied with different numbers of iterations for the number of clusters—from 2 to 200—because no predetermined number of clusters (K) was given. All clustering results were stored and evaluated with three internal cluster validation methods: the silhouette score, the Calinski–Harabasz index, and the Davies–Bouldin index, to determine the optimal number of K for the K-means and the agglomerative hierarchical algorithms using all distances. Figure 2a,b illustrate the scores of each cluster for each clustering validation methods, Silhouette score (a) and Davies–Bouldin index (b), to identify the optimal number of clustering visually. In Figure 2a, a Silhouette score close to 1 implied a better cluster shape. On the contrary, in Figure 2b, a Davies–Bouldin index close to zero implied greater separation between clusters, as described in Section 2.2. 

## 5. Experiment Results

### 5.1. Experiment One: Results

In this experiment, we clustered all the non-operational jobs, taking into account the information provided by the 11 KPIs. With this aim, we applied the k-means algorithm and the agglomerative hierarchical algorithm with different linkage rules, as shown in the experimental set-up in Table 5. We did not have a predetermined number of clusters for both algorithms. The clustering was done with a number of iterations for K from 2 to 200, and the results were fed to the three cluster validation methods to identify the optimal number of clusters.

Table 6 illustrates the comparison of the optimal numbers of clustering for both algorithms using each one of the three validation methods. Regarding the combined selected 11 KPIs job values, we found that the agglomerative hierarchical algorithm performance was better than the K-means algorithm using the Euclidean distance average linkage with a Calinski–Harabasz score of 24,545,720,615 and a silhouette score of 0.523 for three clusters. The combined selected 11 KPIs job values also performed well with the hierarchical single-linkage clustering using the Euclidean distance, with a Davies–Bouldin score of 0.503 for 13 clusters.

### 5.2. Experiment Two: Results

In this experiment, we clustered all the non-operational jobs using only one of the KPIs each time. That is, the study had performed 11 clustering procedures. Once one of the KPIs was selected, the procedure was the same as in experiment one: using the k-means algorithm and the agglomerative hierarchical algorithm with different linkage rules—see the experiment set-up in Table 7. Without a predetermined number of clusters for both algorithms, the number of iterations considered for K ranged from 2 to 200, as was in the previous experiment. Then, the results were fed to the cluster validation methods to identify the optimal number of clusters.

**Figure 3:**
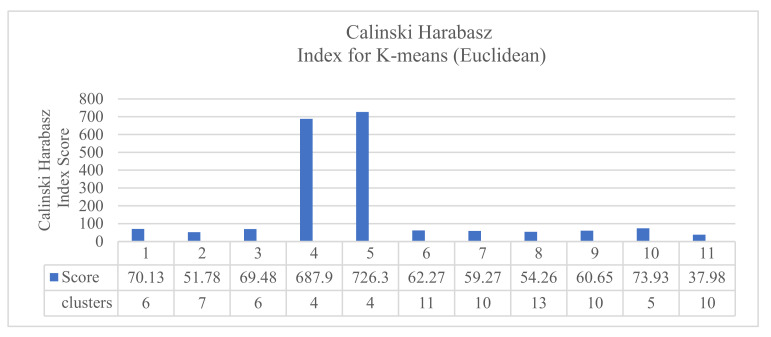
The results of Calinski–Harabasz scores for k-means Euclidean distance.

The results of clustering each of the 11 KPIs individually showed that the K-means performed well using the Euclidean distance. The results gave a Calinski–Harabasz score of 726.341 for four clusters in the KPI interface.bond0.if_octets.tx, as shown in Figure 3.

**Figure 4:**
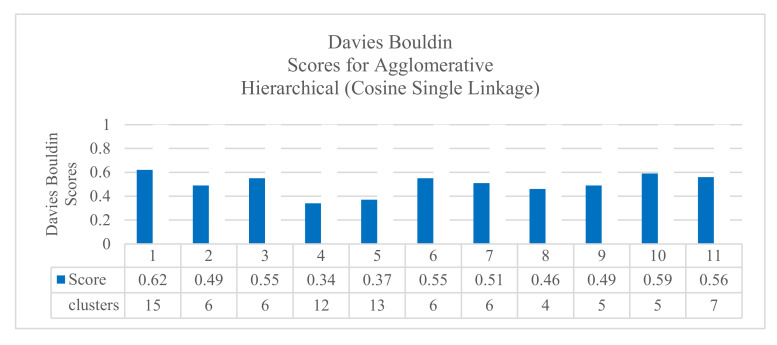
The results of Davies–Bouldin scores for agglomerative hierarchical cosine single linkage.

**Figure 5:**
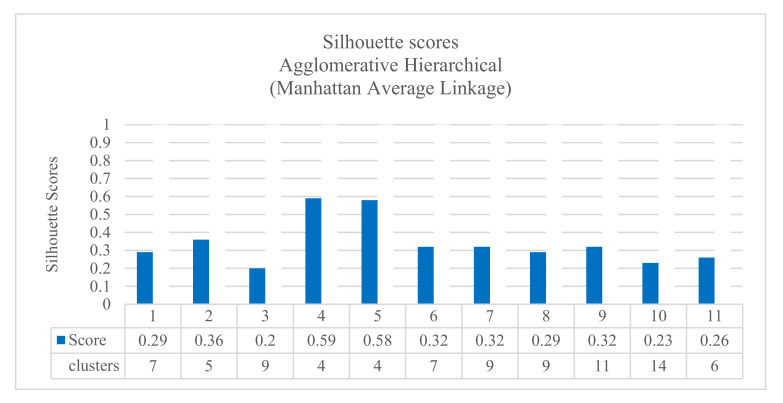
The results of Silhouette scores for agglomerative hierarchical Manhattan average linkage.

Additionally, the results confirmed that the agglomerative hierarchical algorithm performed well in clustering jobs. Figure 4 shows the results with cosine distance, single linkage, and Davies–Boulding index. The results showed a good score (0.340) using the KPI interface.bond0.if_octets.rx with 12 clusters. Figure 5 shows the results with Manhattan distance, average linkage, and Silhouette index. The results showed a good score (0.598) using the KPI interface.bond0.if_octets.rx with four clusters. All the results are summarized in a complete Table (Table A1) in Appendix A.

### 5.3. Validation Experiment

With the aim of validating the conclusions obtained—KPIs belonging to the network interface traffic are the most adequate to obtain a good clustering of non-operational jobs that run in the HPC system—we performed a new experiment with a different dataset also acquired from CESGA. We used the same methodology used in experiments one and two (data preprocessing, data standardization, dimensionality reduction, and clustering), but using only the information about the two selected KPIs: interface.bond0.if_octets.rx and interface.bond0.if_octets.tx.

The dataset stores information about a total amount of 1500 jobs (non-operational jobs), which were running in the 81 available parallel nodes from 1st August 2019 to 31st September 2019. Table 8 shows the number of nodes per job (non-operational) in the new dataset.

The results of clustering based on these two KPIs are shown in Table 9. The highlighted scores in this table demonstrated the best results of the comparison between the scores of the three clustering validation methods for all clustering algorithms. This implied that interface.bond0.if_octets.tx KPI showed better clustering results in all measures—cluster shape, cohesion, and separation—than interface.bond0.if_octets.rx KPI in the performance of both algorithms (K-means and agglomerative hierarchical) with different distance metrics and linkage methods. K-means performed well using the Euclidean distance with Calinski–Harabasz score of 4608.5 for three clusters; the agglomerative hierarchical algorithm performed well in clustering jobs with cosine distance; single linkage of Davies–Boulding score 0.119 for three clusters and Manhattan distance; complete linkage with Silhouette score of 0.858 with three clusters using the KPI interface.bond0.if_octets.rx.

## 6. Discussion

After obtaining the results from both experiments shown in Table 6 and Table A1, we did two comparisons. The first one was done between the results of experiment two to identify which KPI provided the best clustering results in terms of cohesion and separation. With this aim, we analyzed the results obtained from all the experiments that were done, taking into account the information given individually per KPI (different clustering methods, different metrics, different linkage methods, and the assessment with the three quality indexes). The second one was done between the results of experiment one and experiment two to identify which was the best clustering approach, according to the quality indexes. With this aim, we compared the clustering results when we took into account the joint information given by the 11 KPIs together and the results obtained with the KPI that offered the best result in the first comparison.

The results of the first comparison showed that the results obtained by using the KPI interface.bond0.if_octets.rx and the KPI interface.bond0.if_octets.tx were the best ones with different quality indexes. Using the Silhouette score, the KPI interface.bond0.if_octets.rx presented the best clustering results (0.598 for four clusters), followed by the KPI interface.bond0.if_octets.tx (0.580 for four clusters). Using the Davies–Bouldin index, the KPI interface.bond0.if_octets.rx presented the best clustering results (0.34 for 12 clusters), followed by the KPI interface.bond0.if_octets.tx (0.37 for 13 clusters). Using the Calinski–Harabasz index, the KPI interface.bond0.if_octets.tx presented the best clustering results (726.3 for four clusters), followed by the KPI interface.bond0.if_octets.rx (687.9 for four clusters). 

**Figure 6:**
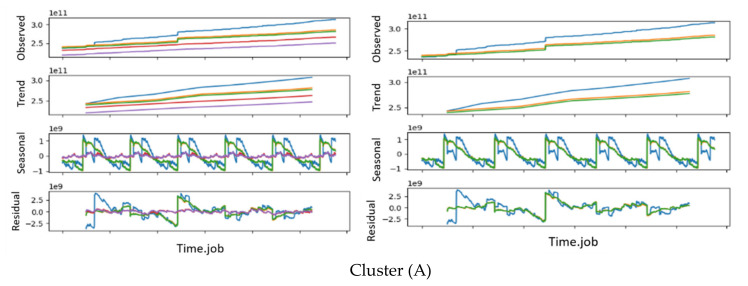

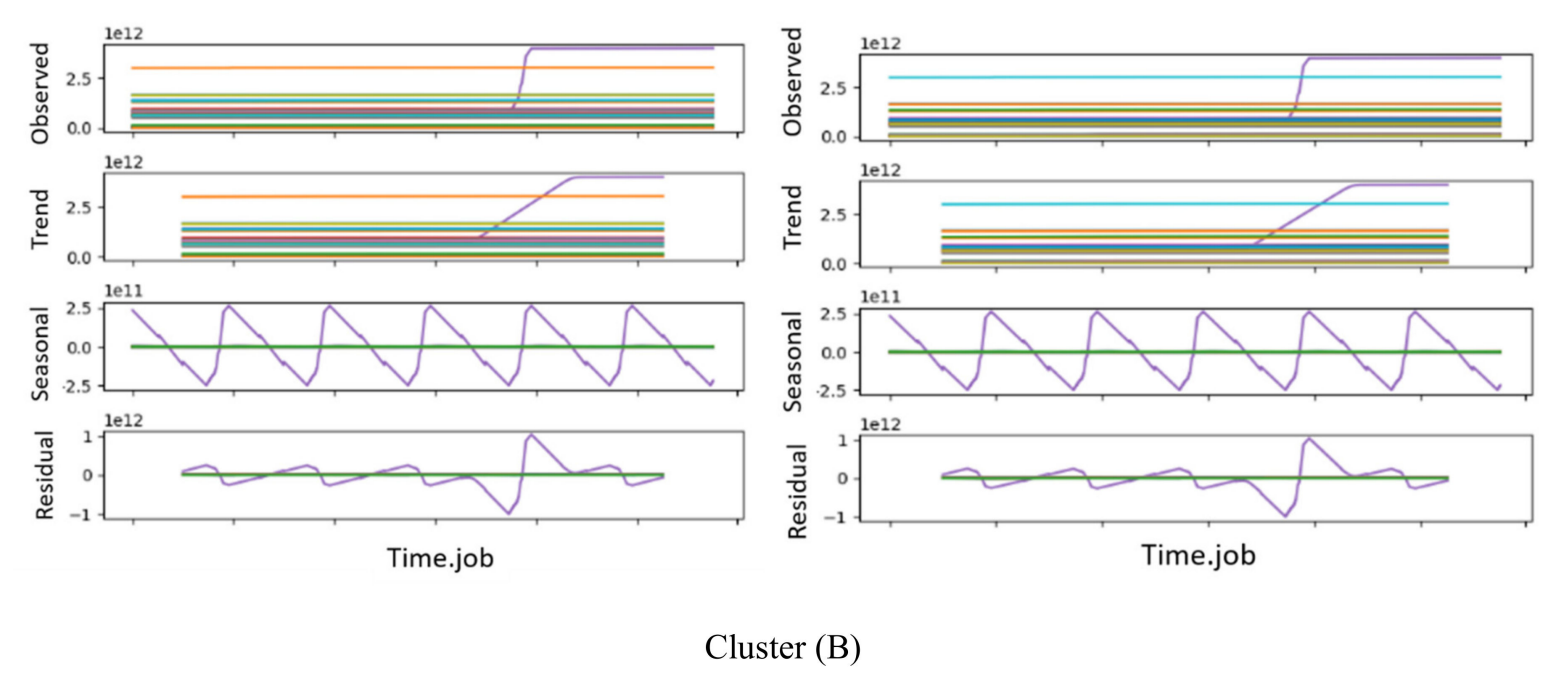
A time series decomposition of jobs from two different clusters of the KPI *interface.bond0.if_octets.rx*: the Cluster (**A**) figure shows the time series decomposition of one job from cluster A, whereas the Cluster (**B**) figure shows the time series decomposition of one job from cluster B.

Consequently, we could conclude that the network (interface) traffic KPIs (interface.bond0.if_octets.rx and interface.bond0.if_octets.tx) presented the best clustering results for all 11 KPIs, providing 4 and 13 clustering, respectively. In order to decide which was the most adequate number of clusters for our dataset, i.e., the most adequate KPI, we analyzed the time series decomposition of all the jobs per cluster. Figure 6 shows sample jobs from two different clusters A and B from the optimal result obtained with the KPI interface.bond0.if_octets.rx. Figure 6 also displays the working nodes’ behaviors of each job. After our analysis, we concluded that this KPI (interface.bond0.if_octets.rx) was the one that showed a high percentage of jobs with similar trends and behavior. 

The results of the second comparison concluded that according to the Silhouette and Davies–Bouldin indexes, the best results were obtained by applying hierarchical algorithms. However, and according to the Calinski–Harabasz index, K-means was the best option. Since we obtained the same conclusion in two out of three clustering validation methods, we considered that a hierarchical algorithm was the most adequate for our purpose. Besides, the Calinski–Harabasz index did not have an upper-value level, so it was usually applied to compare different classifications with the same conditions, which reinforced our approach.

Finally, our results were validated by conducting a clustering experiment with a new dataset, which confirmed that the network (interface) traffic KPIs (interface.bond0.if_octets.rx and interface.bond0.if_octets.tx) showed the best clustering results.

## 7. Conclusions

This study aimed to provide a methodology to cluster HPC jobs (non-operational) in order to automatically detect different types of jobs according to their performance. The job performance could be studied by using the KPI metrics provided by the HPC monitoring system. Our goal was also to select the most suitable or representative set of KPIs for clustering non-operational jobs according to their performance. Our analysis and validation were done by using a dataset provided by the Supercomputing Center of Galicia (CESGA) that collected the information of the KPIs of 1783 jobs from 1 June 2018 to 31 July 2018.

Considering a large amount of available KPIs (44,280), we made a previous selection based on the advice from experts who work at CESGA. They provided us with 11 KPIs from the following categories: CPU usage, memory usage, IPMI, system load, and network (interface) traffic.

We performed two different kinds of experiments in order to select the most suitable KPIs for clustering HPC jobs. The first experiment performed the clustering by combining the information gathered from the 11 KPIs, whereas the second one performed the individual clustering individually for each one of the 11 KPIs. Both experiments were done by using different clustering algorithms (K-means and agglomerative hierarchical algorithm), using different linkage methods (single-linkage, complete-linkage, average-linkage, and Ward’s method) and using different distance metrics (Euclidean, Manhattan, and Cosine). In order to assess the quality of the obtained clusters, we also used different indexes (Silhouette, Calinski–Harabasz, and Davies–Boulding). Before performing the clustering, we applied PCA in order to reduce the dimensionality of the data, without losing information, to reduce the computational load of the algorithms. Finally, a clustering experiment, based only on the two selected KPIs (interface.bond0.if_octets.rx and interface.bond0.if_octets.tx), was performed, with the aim of validating our approach. For this, we obtained a new dataset with 1500 jobs (non-operational) from 1 August 2019 to 31 September 2019. The results confirmed our proposal.

Our analysis concluded that the clustering based on the joint information given by the 11 KPIs performed worse than the clustering based on the individual KPIs. What is more, the results showed that the information given by those KPIs belonging to the network (interface) traffic was the most adequate (interface.bond0.if_octets.rx and interface.bond0.if_octets.tx). The clusters obtained with the information of these KPIs showed the best quality in terms of cohesion and separation of HPC jobs. More specifically, the visualization of the KPI (interface.bond0.if_octets.rx) clusters showed a high percentage of jobs with similar trends. Therefore, our methodology could be applied to any data set with information about these two KPIs in order to obtain a good clustering and infer the number of types of non-operational jobs that run in the HPC system. The procedure is simple and offers a solution to some challenges faced in other experimentations [9,10,11] when dealing with similar unlabeled data with the large dimensionality.

In our opinion, this clustering phase should be considered the first stage in a broader procedure to detect anomalies in HPC systems. In fact, we are currently working on analyzing this categorization. We consider that the obtained clusters would help to infer similar characteristics of the jobs belonging to each cluster that, definitively, could give information to detect those jobs whose performance is not the expected one and be able to early detect potential anomalies in the system. Finally, and although we have checked that the mechanism applied for dimensionality reduction (fixed PCA) supports a good percentage of retained information, we are working to improve this aspect. Since it was mentioned in the literature [25,27], the problem with the cost function used in PCA entails that there are retained large pairwise distances instead of focusing on retaining small pairwise distances, which is usually much more important. The solution given in [25] is defining a specific cost function based on a non-convex objective function. We are currently defining this new cost function using a larger dataset obtained from the same high performance computing center. We are also considering using KPIs time series feature extraction in our clustering methodology. The extracted features of statistical significance will be evaluated and analyzed by different state-of-art machine learning approaches to achieve our purpose.

## Figures and Tables

**Table 1:** Performance metrics selected.

Category	Metric	Definition
CPU usage	aggregation.cpu-average.percent.idle	The aggregated average percent of the time when the CPU is idle.
	aggregation.cpu-average.percent.system	The aggregated average percent of the time when the CPU is working.
	aggregation.cpu-average.percent.wait	The aggregated average percent of the time when the CPU is waiting.
Network (interface) traffic	interface.bond0.if_octets.rx interface.bond0.if_octets.tx	The number of bytes received over the network per second. The number of bytes transmitted over the network per second.
IPMI (intelligent platform management interface)	ipmi.CPU1_Temp ipmi.CPU2_Temp ipmi.PW_consumption ipmi.System_Temp	The temperature readings of CPU1. The temperature readings of CPU2. The power consumed by the system hardware. The temperature readings of the system.
System load	load.load.shortterm	System load average over the last minute.
Memory usage	memory.cached.memory	Cached memory occupied.

**Table 2:** Basic data analysis.

Tota Number of Jobs		1783
Jobs	Operational	1281
	Non-operational	302
Jobs excluded because they contained less than two nodes		144
Jobs excluded because they were not included in all the 11 KPI (key performance indicator) matrices		56

**Table 3:** The number of nodes per job (non-operational).

Node Ranges	Number of Jobs
2–5	195
6–10	49
11–15	15
16–20	13
>20	30
Total	302

**Table 4:** Principal component analysis (PCA)—two principal components retained information.

Retained Information Ranges Percentages	Number of Jobs
>=95%	142
>=90%–<95%	76
>=85%–<90%	27
>=80%–<85%	26
>=75%–<80%	20
<75%	11

**Table 5:** Experiment one set-up.

Number of Jobs	Clustering Algorithm	Cluster Validation Methods
302 non-operational jobs	K-means, agglomerative hierarchical	Silhouette score
		Calinski–Harabasz index
		Davies–Bouldin index

**Table 6:** Experiment one: results.

				Calinski–Harabasz Index	Davie–Bouldin Index	Silhouette Score
**K-mean**	**Euclidean**		**K**	8	6	6
			**Score**	14,591,690,919	1.399	0.313
**Agglomerative hierarchical**	**Cosine**	**Average**	**K**	8	11	8
			**Score**	11,439,074,172	1.721	0.236
		**Complete**	**K**	14	12	11
			**Score**	6,395,676,953	1.916	0.176
		**Single**	**K**	3	8	3
			**Score**	22,147,812,202	1.173	0.074
	**Euclidean**	**Ward**	**K**	4	4	4
			**Score**	22,147,812,202	0.961	0.069
		**Average**	**K**	**3**	9	**3**
			**Score**	**24,545,720,615**	0.607	**0.523**
		**Complete**	**K**	8	24	8
			**Score**	14,665,419,924	1.414	0.288
		**Single**	**K**	12	**13**	13
			**Score**	6,787,242,293	**0.503**	0.368
	**Manhattan**	**average**	**K**	13	12	13
			**Score**	8,253,129,705	0.726	0.384
		**complete**	**K**	4	4	4
			**Score**	19,996,681,144	1.193	0.314
		**single**	**K**	16	9	16
			**Score**	15,670,931,203	0.5933	0.270

**Table 7:** Experiment two set-up.

KPI	302 operational Jobs Considering Each KPI Individually	Clustering Algorithm	Cluster Validation Methods (VM)
1	aggregation.cpu-average.percent.idle	K-means, agglomerative hierarchical	Calinski–Harabasz index (C)
2	aggregation.cpu-average.percent.system		
3	aggregation.cpu-average.percent.wait		Davies–Bouldin index (D)
4	interface.bond0.if_octets.rx		
5	interface.bond0.if_octets.tx		
6	ipmi.CPU1_Temp		Silhouette score (S)
7	ipmi.CPU2_Temp		
8	ipmi.PW_consumption		
9	ipmi.System_Temp		
10	load.load.shortterm		
11	memory.cached.memory		

**Table 8:** The number of nodes per job (non-operational) in the validation experiment’s new dataset.

Node Ranges	Number of Jobs
2–5	856
6–10	320
11–15	74
16–20	56
>20	194
Total	1500

**Table 9:** Validation experiment: results.

				interface.bond0.if_octets.rx			interface.bond0.if_octets.tx		
				Calinsk–Harabasz Index	Davie–Bouldin Index	Silhouette Score	Calinski–Harabasz Index	Davie–Bouldin Index	Silhouette Score
**K-mean**	**Euclidean**		**K**	2	2	2	3	2	5
			**Score**	2935.4	0.6332	0.6545	**4608.5**	0.331	0.779
**Agglomerative hierarchical**	**Cosine**	**Average**	**K**	4	2	4	3	3	3
			**Score**	1893.5	0.3284	0.6636	2321.4	0.138	0.8548
		**Complete**	**K**	5	2	5	4	2	3
			**Score**	1764.8	0.3284	0.6508	2268.6	0.149	0.8562
		**Single**	**K**	5	2	17	4	3	3
			**Score**	747.9	0.3284	0.6242	2336.4	**0.119**	0.8508
	**Euclidean**	**Ward**	**K**	2	3	3	2	2	6
			**Score**	2882.2	0.6399	0.6627	4569.9	0.3589	0.7810
		**Average**	**K**	**4**	2	**3**	**12**	**2**	**3**
			**Score**	2010.0	0.3284	0.6532	3116.9	0.1492	0.8563
		**Complete**	**K**	11	2	3	2	2	3
			**Score**	2603.9	0.3284	0.6502	2351.0	0.1492	0.85841
		**Single**	**K**	7	**2**	8	4	3	3
			**Score**	1274.4	0.3284	0.6176	2336.4	0.1199	0.85088
	**Manhattan**	**Average**	**K**	5	2	3	12	2	3
			**Score**	2010.0	0.3284	0.6238	3122.1	0.1492	0.85634
		**Complete**	**K**	8	2	3	2	2	3
			**Score**	2143.8	0.3284	0.6365	2351.0	0.1492	**0.85848**
		**Single**	**K**	8	2	8	4	3	3
			**Score**	1221.9	0.3284	0.61760	2336.4	0.1199	0.8508

## Appendix A

**Table A1:** Experiment two: results.

KPI	VM	K-Means		Agglomerative Hierarchical																			
		Euclidean		Euclidean								Cosine						Manhattan					
				Average		Complete		Single		Ward		Average		Complete		Single		Average		Complete		Single	
		K	Score	K	Score	K	Score	K	Score	K	Score	K	Score	K	Score	K	Score	K	Score	K	Score	K	Score
1	C	6	70.13	5	53.53	12	28.02	16	11.59	11	13.89	10	32.07	8	51.30	15	15.84	8	37.34	14	41.21	7	4.98
	D	8	1.59	5	1.940	11	1.99	5	1.34	10	1.45	9	0.78	5	1.11	15	0.62	7	0.77	7	1.46	8	0.62
	S	9	0.251	7	0.137	5	0.14	16	0.034	9	0.24	10	0.24	8	0.20	15	0.12	7	0.29	7	0.19	8	0.18
2	C	7	51.78	11	22.05	20	22.73	11	10.02	6	23.68	15	21.85	5	52.12	6	29.01	5	36.48	7	34.92	22	9.65
	D	4	1.40	17	1.57	11	2.08	10	1.32	3	1.37	11	0.61	10	1.08	6	0.49	5	0.78	10	1.34	7	0.502
	S	4	0.212	11	0.12	11	0.10	11	-0.07	8	0.21	15	0.23	5	0.21	6	0.38	5	0.36	8	0.18	22	0.02
3	C	6	69.48	6	22.85	5	25.75	6	22.56	4	33.21	13	35.21	3	90.84	7	33.48	5	66.19	14	32.55	4	57.69
	D	5	1.347	11	1.94	8	2.28	6	1.50	5	1.10	9	0.99	20	1.09	6	0.55	7	0.97	5	1.31	6	0.75
	S	5	0.254	13	0.11	5	0.09	12	0.03	5	0.25	13	0.25	6	0.25	7	0.25	9	0.20	5	0.19	6	0.08
4	C	4	687.9	9	92.54	9	111.2	9	84.32	6	110.3	13	341.4	6	90.5	11	184.7	4	615.8	8	366.6	17	151.4
	D	8	0.96	4	1.02	12	1.92	6	0.85	8	0.92	9	0.74	7	0.80	12	**0.34**	9	0.46	30	0.83	22	0.37
	S	8	0.39	12	-0.02	9	0.024	9	0.01	8	0.40	9	0.53	7	0.48	11	0.45	4	**0.59**	6	0.53	17	0.39
5	C	4	**726.3**	18	58.29	14	86.9	6	66.5	6	77.8	11	366.4	4	638.6	6	300.5	5	514.1	6	600.2	3	440.5
	D	8	1.04	19	1.00	29	1.22	18	0.83	6	1.09	6	0.56	4	0.603	13	0.37	6	0.55	7	0.96	5	0.53
	S	8	0.37	10	0.007	6	0.01	9	0.01	6	0.351	4	0.70	4	0.56	6	0.39	4	0.58	7	0.387	5	0.56
6	C	11	62.27	6	34.98	15	21.92	3	45.88	5	30.42	6	54.48	9	62.28	12	25.59	8	58.50	9	59.09	4	59.33
	D	4	1.09	6	1.88	9	2.15	4	1.13	4	1.10	13	0.74	5	1.00	6	0.55	4	0.60	4	0.89	4	0.49
	S	4	0.28	6	0.07	7	0.35	5	0.21	4	0.28	6	0.31	5	0.30	5	0.31	7	0.32	9	0.24	4	0.32
7	C	10	59.27	6	38.27	7	22.71	4	45.52	5	50.4	7	46.62	11	51.31	5	45.19	6	60.70	10	53.21	6	38.22
	D	4	1.63	9	1.57	8	1.97	6	1.42	4	1.03	19	0.70	6	0.94	6	0.51	9	0.72	5	0.87	9	0.54
	S	4	0.31	7	0.06	5	0.06	5	-0.03	4	0.296	7	0.33	11	0.25	5	0.31	9	0.32	7	0.26	6	0.30
8	C	13	54.26	4	61.43	16	28.88	8	27.04	11	60.86	7	46.49	5	76.66	4	59.1	10	47.57	10	52.39	9	24.82
	D	9	1.51	7	1.66	7	2.31	6	1.17	4	1.05	17	0.74	12	1.02	4	0.46	18	0.74	11	1.05	9	0.54
	S	4	0.29	9	0.11	7	0.08	4	0.008	4	0.29	7	0.32	11	0.25	4	0.37	9	0.294	10	0.26	9	0.23
9	C	10	60.65	5	43.82	7	49.37	14	16.54	11	56.54	11	40.54	5	82.12	5	34.38	11	38.15	6	78.59	7	24.53
	D	13	1.39	7	1.21	7	2.12	4	1.29	6	1.09	6	0.684	5	0.99	5	0.49	13	0.76	4	0.97	7	0.50
	S	4	0.34	5	0.05	9	0.17	14	0.009	6	0.319	11	0.32	5	0.34	5	0.34	11	0.32	5	0.34	7	0.28
10	C	5	73.93	5	45.75	4	50.93	8	17.27	10	56.25	6	38.23	6	62.1	17	14.04	9	35.37	13	39.82	6	4.25
	D	5	1.71	6	2.03	5	1.96	9	1.40	5	1.71	5	0.74	22	1.21	5	0.59	6	0.74	5	1.27	6	0.58
	S	6	0.19	5	0.088	5	0.13	5	-0.04	9	0.23	6	0.26	7	0.16	7	0.19	14	0.23	5	0.20	4	0.21
11	C	10	37.98	5	36.32	10	17.75	8	11.60	10	40.35	10	21.83	8	35.19	5	38.38	8	25.13	9	31.11	18	11.71
	D	9	1.83	6	1.99	8	2.19	15	1.15	5	1.27	4	0.57	5	1.21	7	0.56	4	0.57	8	1.72	4	0.55
	S	5	0.16	5	0.13	4	0.07	13	-0.03	4	1.18	7	0.28	5	0.30	5	0.31	6	0.26	4	0.13	9	0.12
